# Meta-Analysis of High-Throughput Datasets Reveals Cellular Responses Following Hemorrhagic Fever Virus Infection

**DOI:** 10.3390/v3050613

**Published:** 2011-05-12

**Authors:** Gavin C. Bowick, Alexander J. McAuley

**Affiliations:** 1 Department of Microbiology & Immunology, Institute for Human Infections & Immunity, University of Texas Medical Branch, Galveston, TX 77555, USA; E-Mail: ajmcaule@utmb.edu; 2 Center for Biodefense and Emerging Infectious Diseases, Sealy Center for Vaccine Development, University of Texas Medical Branch, Galveston, TX 77555, USA

**Keywords:** viral hemorrhagic fever, arenavirus, Ebola virus, Rift Valley fever virus, microarray, proteomics, bioinformatics

## Abstract

The continuing use of high-throughput assays to investigate cellular responses to infection is providing a large repository of information. Due to the large number of differentially expressed transcripts, often running into the thousands, the majority of these data have not been thoroughly investigated. Advances in techniques for the downstream analysis of high-throughput datasets are providing additional methods for the generation of additional hypotheses for further investigation. The large number of experimental observations, combined with databases that correlate particular genes and proteins with canonical pathways, functions and diseases, allows for the bioinformatic exploration of functional networks that may be implicated in replication or pathogenesis. Herein, we provide an example of how analysis of published high-throughput datasets of cellular responses to hemorrhagic fever virus infection can generate additional functional data. We describe enrichment of genes involved in metabolism, post-translational modification and cardiac damage; potential roles for specific transcription factors and a conserved involvement of a pathway based around cyclooxygenase-2. We believe that these types of analyses can provide virologists with additional hypotheses for continued investigation.

Viral hemorrhagic fevers represent an important group of emerging infections: Lassa virus is an important public health problem in West Africa and Ebola virus causes sporadic outbreaks associated with high mortality. The pathogenesis of hemorrhagic fevers may involve aberrant immune responses leading to the dysregulation of cytokines production and vascular leakage. The immune responses to hemorrhagic fevers have been extensively studied, with networks based around interferon shown to be particularly important. However, the roles of other signaling pathways, both immune and non-immune, have not been characterized as thoroughly. In particular, there is relatively little information on the effect on pathways involved in basic cellular metabolism and homeostasis. Given the tropism of many of these viruses for mononuclear phagocytes and the central role of these cells in mediating immune responses, dysregulation of basic function may be an important contributor to pathogenesis.

The increase in the use of unbiased high-throughput methods, such as transcriptomic microarrays and proteomics, has provided a resource of large datasets, which reveal the myriad ways the host-cell responds to viral infection. These experiments have, in some ways, been victims of their own success. The large number of differentially expressed genes or proteins means that not all differentially expressed genes can be validated. Whole datasets are visualized using techniques such as hierarchical clustering, with validation often limited to the genes showing the largest fold change, or those which are hypothesized to be involved in pathogenesis, such as immune response genes. More recently, network analysis has been used to place high-throughput datasets into more of a functional context and show expression changes of multiple genes in the same pathway [1]. Gene ontology analysis has been used to show the expression of genes in particular functional classes, and promoter enrichment can be used to identify which transcription factors may be centrally involved in controlling gene expression [2,3].

The understanding of the role of cell signaling pathways may have important implications for the development of novel antiviral therapies. Inhibiting cellular proteins required for replication or, particularly in the case of the hemorrhagic fevers, modulating pathways that lead to immunopathology or cellular dysregulation may prove to be an effective therapeutic strategy with a lower risk of the evolution of resistance compared to inhibiting viral proteins. As an example, a DNA aptamer containing the AP-1 consensus sequence has been shown to protect guinea pigs against lethal arenavirus challenge [4]. This type of therapeutic strategy may also prove to be effective against several viruses that exploit or manipulate similar cellular pathways.

We have previously used network analysis to investigate cellular responses to Pichindé virus (PICV) infection [5–8]. In this note, we have used network analysis tools to compare cellular responses in hemorrhagic fever systems using meta-analysis of published high-throughput datasets. We believe that these types of approaches will become increasingly relevant as the number of datasets continues to grow, and will play a central role in allowing the development of hypotheses regarding the cellular response to viral infection that are focused on under-researched pathways in the field.

We created gene/protein lists derived from previously published high-throughput datasets: microarray expression data from Ebola virus (EBOV) [9], lymphocytic choriomeningitis virus (LCMV) models of Lassa fever [10,11] (from both PBMCs and the liver), Rift Valley fever virus (RVFV) [12] and a proteomics dataset from a PICV model of Lassa fever [8]. Where possible, these lists were constructed from those genes/proteins that were differentially expressed between attenuated and virulent infection.

We began our analysis by uploading the datasets to the Ingenuity Pathway Analysis application [13] and looking at an overview of the dataset in the form of enrichment of specific genes associated with canonical cell signaling pathways, functions and diseases. As expected, the LCMV datasets showed an involvement in many more pathways than the EBOV, RVFV and PICV datasets due to the significantly higher number of genes in the datasets. For this reason, we chose not to focus on pathways which did not include RVFV, EBOV or PICV. Pathogenic functions identified in RVFV predicted high-significance of hepatic and renal disease, observed in clinical cases [14]. We also identified potential involvement of EBOV in mediating expression changes of genes associated with cardiac damage, suggested to be involved in EBOV infection [15]. Genes involved in regulating post-translational modification (PTM) and metabolic pathways including oxidative phosphorylation, lysine degradation, tyrosine metabolisms and ubiquinone synthesis showed enrichment following arenavirus infection, in the PICV and LCMV liver datasets in particular. The relevance for these pathways in infection is unclear, however changes in the ability to post-translationally modify proteins could have significant effects on many cell signaling pathways; changes in basic metabolism could alter homeostasis, reducing the capacity of infected cells to respond to stimuli appropriately.

A further way to interpret microarray data is to consider the differentially expressed genes as the observed outcome of upstream cell signaling events that may be activated or inhibited by virus infection. By utilizing databases of genome sequence data, we investigated whether any particular transcription factor binding sites were associated with the observed transcriptional changes to a greater degree than would be expected by chance. We used the PSCAN web server [16] to identify the five most significant transcription factors, which may act as ‘master regulators’ in controlling transcription in response to infection (Table 1). We selected a region 950 base pairs upstream and 50 base pairs downstream of the translational start site for analysis using the TRANSFAC database [17]. Few significant transcription factors were identified in the EBOV dataset, with none of the *p* values being significant after Bonferroni correction for multiple hypothesis testing. We speculate that part of the reason for this is that the dataset is based on a comparison between infection with wild-type and inactivated virus; we hypothesize that both of these infections stimulate similar innate pathogen recognition receptors and lead to a number of conserved downstream gene expression changes.

Analysis of the RVFV dataset revealed enrichment of promoters for the interferon-inducing transcription factors IRF7 and IRF2, consistent with the central role of interferon induction pathways in infection and in agreement with the results reported by the authors in the original study [12]. Of particular interest is the group of transcription factors predicted to be master regulators of the responses to LCMV infection of PBMCs. We also observed enrichment of binding sites for the transcription factor SP1, which we had previously identified in networks built following PICV infection, and which showed differential DNA-binding activities between infection with attenuated and virulent viruses early during *in vitro* infection [6].

We next created a network using the Ingenuity Pathways Analysis tool using the implicated transcription factors as starting point, we sought to identify the upstream signaling pathways that may be involved in regulating their transactivation activities. Using interactions mined from the literature, we identified the mitogen-activated protein kinase pathway, p38 in particular, in controlling responses to LCMV infection (Figure 1). This is consistent with previously published data that shows activation of these signaling molecules and transcription factors in response to PICV infection [18,19].

Combining bioinformatics-based pathway analysis with gene expression data allows for the discovery of cellular pathways and possible interactions that may be overlooked when considering the data in a tabular format. Following analysis of RVFV expression data in the form of predicted significance of canonical pathways and functions, we identified a network based around cyclooxygenase-2 (COX-2). Overlaying expression data from the microarray revealed downregulation of COX-2 expression, despite upregulation of IL-6 and STAT1, both of which have been shown to induce COX-2 expression (Figure 2). Interestingly, COX-2 has also been implicated in other hemorrhagic fever infections and models, showing downregulation in the LCMV macaque model [11], upregulation in both EBOV and Dengue infection [20,21]. The conserved identification of changes in COX-2 expression and activation of its regulatory networks suggest that this might be a potential target for further investigation.

We have used web-based bioinformatics applications to investigate cellular responses to infection with hemorrhagic fever viruses. As additional datasets become available, our ability to investigate common and specific responses to viral infection will continue to grow, particularly as additional bioinformatics analysis tools and databases are developed. In particular, as the number of datasets describing models of varying pathogenesis increases, we will be better placed to perform comparative analyses to define the cellular responses associated with attenuation, facilitating rational vaccine design [22]. In this report, we have shown how currently available bioinformatics tools can be used to leverage the additional data from high-throughput studies to generate additional hypotheses for further testing. These types of analyses may provide additional clues toward understanding how hemorrhagic fever virus infection perturbs cellular functions and playing a role in uniting virus-host interactions at the molecular and cellular level with the mechanisms which underlie clinical pathology.

## Figures and Tables

**Figure 1:**
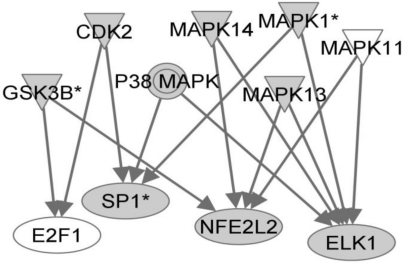
Signaling pathways upstream of predicted transcriptional regulators in lymphocytic choriomeningitis virus (LCMV) infection of PBMCs. The Ingenuity Pathways Analysis application was used to search for proteins known to be involved in controlling the activity of transcription factors that bind to motifs identified using PSCAN analysis. Shaded proteins were shown to have differential expression by microarray analysis.

**Figure 2:**
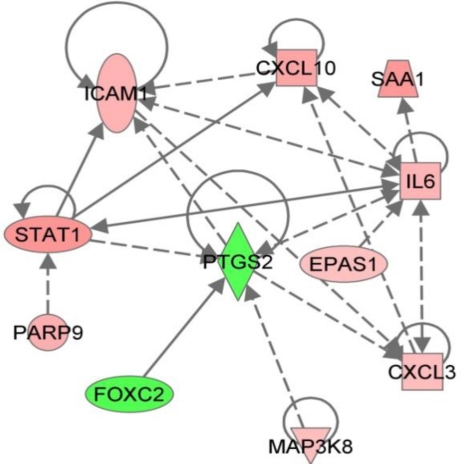
Regulation of a signaling network based around cyclooxygenase-2 (PTGS2). The Ingenuity Pathways Analysis application was used to construct a network on the basis of known protein and gene interactions, expression data from the Rift Valley fever microarray study is overlayed: red indicates upregulation, green indicates downregulation.

**Table 1:** Top five predicted transcription factor binding motifs identified using the PSCAN algorithm; *p* values are indicated and motifs shown in bold are those which remained significant following Bonferroni adjustment for multiple comparisons.

**EBOV**	**RVFV**	**LCMV (PBMC)**	**LCMV (LIVER)**
FAC1 (0.017)	**ISRE1** (1.2 × 10^−15^)	**SP1** (1.2 × 10^−22^)	**E2F** (2.8 × 10^−18^)
NFY (0.018)	**IRF7** (2.4 × 10^−10^)	**E2F** (3.9 × 10^−16^)	**ELK1** (5.6 × 10^−17^)
ATF6 (0.021)	**IRF2** (3.6 × 10^−6^)	**ELK1** (2 × 10^−14^)	**SP1** (3.7 × 10^−15^)
FOXO4 (0.021)	**AP1** (1.3 × 10^−5^)	**EGR3** (8.7 × 10^−12^)	**ATF** (3.5 × 10^−11^)
RFX1 (0.022)	NF-κB (3 × 10^−4^)	**NRF2** (9.4 × 10^−11^)	**AP2** (6.3 × 10^−11^)
